# Insufficient support and help for adolescents with disabilities during the COVID-19 pandemic: Marja Eliisa Holm

**DOI:** 10.1093/eurpub/ckac131.468

**Published:** 2022-10-25

**Authors:** ME Holm, P Sainio, S Sjöblom, P Nurmi-Koikkalainen

**Affiliations:** Department of Public Health and Welfare, Finnish Institute for Health and Welfare, Helsinki, Finland

## Abstract

**Background:**

The COVID-19 pandemic may have increased the need for help and support for adolescents with disabilities. At the same time, moving to distance learning can reduce access to support services. Despite this, little is known about this issue. Thus, we investigated differences in the unmet need for help and support between adolescents with and without disabilities during the COVID-19 pandemic.

**Methods:**

We analyzed large population-based data from the Finnish School Health Promotion study obtained during 2019 and 2021 (2019: n = 87,215; 2021: n = 91,560). The target group comprised adolescents from lower secondary schools in Finland (age: M = 15.3, SD = .64). Logistic regression models were applied to investigate differences in the unmet need for help and support between adolescents with and without disabilities.

**Results:**

During the pandemic, adolescents with disabilities reported insufficient help and support related to their learning and well-being from teachers, school curators and psychologists, and school nurses and doctors more often than other adolescents (p < .001). Adolescents with disabilities reported more often than others that distance learning involved insufficient learning support (p < .001). Between 2019 and 2021, an increasing proportion of adolescents with disabilities felt that they had received insufficient help and support related to their well-being from nurses, doctors, psychologists, school curators, and teachers. To summarize, the COVID-19 pandemic reduced access to support and assistance for adolescents, particularly those with disabilities.

**Conclusions:**

Policies in schools should be developed and resources secured so that support and help for adolescents with disabilities can be secured in exceptional circumstances. Insufficient support and assistance for adolescents with disabilities can impair their learning outcomes and health.

**Key messages:**

• During the COVID-19 pandemic, adolescents with disabilities reported insufficient help and support related to their learning and well-being more often than other adolescents.

• In times of crisis, support and help for adolescents with disabilities must be guaranteed.

